# Impact of Lockdown Measures on Joint Music Making: Playing Online and Physically Together

**DOI:** 10.3389/fpsyg.2021.642713

**Published:** 2021-05-28

**Authors:** Kelsey E. Onderdijk, Freya Acar, Edith Van Dyck

**Affiliations:** ^1^IPEM, Department of Art, Music and Theatre Sciences, Ghent University, Ghent, Belgium; ^2^Department of Data Analysis, Ghent University, Ghent, Belgium

**Keywords:** music, lockdown, social bonding, togetherness, COVID-19, joint music making, connectedness

## Abstract

A wide range of countries decided to go into lockdown to contain the coronavirus disease (COVID-19) pandemic of 2020, a setting separating people and restricting their movements. We investigated how musicians dealt with this sudden restriction in mobility. Responses of 234 people were collected. The majority of respondents (95%) resided in Belgium or the Netherlands. Results indicated a decrease of 79% of live music making in social settings during lockdown compared with before lockdown. In contrast, an increase of 264% was demonstrated for online joint music making. However, results showed that most respondents were largely or even completely unaccustomed with specialized platforms for online joint music making (e.g., JamKazam, Jamulus). Respondents reported to mostly use well-known video-conferencing platforms such as Zoom and Skype when playing together virtually. However, when such video-conferencing platforms were used, they were often not employed for synchronized playing and were generally reported to insufficiently deal with latency issues. Furthermore, respondents depending on music making as their main source of income explored online real-time methods significantly more than those relying on other income sources. Results also demonstrated an increase of 93% in the use of alternative remote joint music-making methods (e.g., recording parts separately and subsequently circulating these digital recordings). All in all, results of this study provide a more in-depth view on joint music making during the first weeks of lockdown induced by the COVID-19 pandemic of 2020, and demonstrate users’ perceptions of performance and usability of online real-time platforms as well as alternative methods for musical interaction.

## Introduction

On March 11, 2020, the World Health Organization classified the outbreak of the 2019 novel coronavirus disease (COVID-19) as a pandemic (WHO, 2020b). Several European and other countries decided to go into lockdown for an undetermined amount of time, as evolutions of the situation were hard to predict. This unprecedented setting separated people and restricted their movements. In Belgium, the National Security Council (CNS) decided that, from March 13 onward, people were only allowed to leave their residences for essential reasons (e.g., going to work if telework was not possible, going out for a doctor’s appointment, going to the stores) and for outdoor activities that did not involve physical contact. Such activities could only take place with one other individual, and at all times, social distancing measures had to be applied (i.e., maintaining at least 1.50 m between non-cohabitating individuals) (Diplomacy Belgium, 2020).

Due to these restrictions, all music and dance events with live audiences were prohibited, resulting in the cancelation of music concerts and festivals, and the temporary closure of nightclubs. Correspondingly, a range of initiatives rapidly emerged, attempting to provide virtual alternatives to such events. Some music concerts and festivals shifted to online platforms, where they were often livestreamed through global social media or video platforms such as Facebook, Twitch, or YouTube (Thomas, 2020). Moreover, virtual raves became popular means for people to socialize and/or experience live DJ performances in compliance with social distancing principles. Such events were often broadcasted live to audiences ranging from small groups of individuals up to thousands or even millions of people around the world through online platforms (Palamar and Acosta, 2020; Ren, 2020; Weaver et al., 2020).

On a smaller scale, methods and settings employed by musicians to simply play, jam, and/or rehearse with others had to be rethought as well. Taking the above-described lockdown restrictions into account, live interaction was only allowed between two individuals maximally. Moreover, such events could only take place outdoors, and at all times, a distance of at least 1.50 m had to be maintained. It seems evident that these restrictions impacted joint music making, prompting the current study to investigate to what extent musicians continued to play music with others, as well as the methods they used to do so.

**FIGURE 1:**
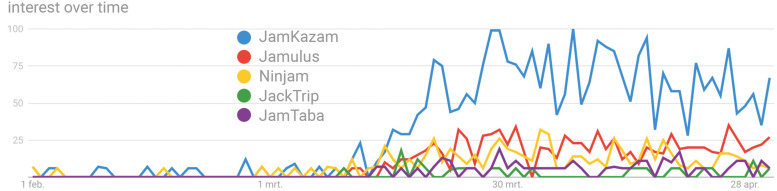
The figure illustrates the interest over time in specialized joint music-making platforms based on global Google searches. The time frame is provided on the *x*-axis (i.e., February 1 to May 1). Numbers on the *y*-axis represent search interest relative to the highest point (i.e., 100). Five (i.e., maximum input) of the most popular platforms are shown. For many countries, lockdown measures took effect in March, from which on we can see that an increase in interest arose. Data source: Google Trends (https://www.google.com/trends).

It has been well established that music is an inherently social activity; throughout history and in almost all cultures, music has been functioning as a means to create and consolidate social bonds (Nettl, 1983, 2000; Roederer, 1984; McNeill, 1995; Freeman, 2000; Dunbar, 2004). Increasingly, research on joint music making has been focusing on this bonding capacity. For example, it was demonstrated that by playing music with others, a sense of group identity can be obtained and positive intergroup attitudes can be developed (Laiho, 2004; Bakagiannis and Tarrant, 2006; Kirschner and Tomasello, 2010; Miranda and Gaudreau, 2011; Rabinowitch et al., 2013; Cirelli et al., 2014; Volpe et al., 2016). Indeed, during the current pandemic, people engaged in musical activities for social components. So-called balcony concerts and initiatives such as WHO’s “One World: Together At Home” consisted of expressions of social solidarity and feelings of togetherness (Taylor, 2020; WHO, 2020a). The question remains whether musicians were able to maintain meaningful interactions in joint music-making settings and to what extent social connectedness was of importance herein. Due to the lockdown regulations, possible alternatives consisted of live music making with cohabitants or–yet only outdoors–with maximally one non-cohabitating individual or by using virtual methods to play with fellow musicians. The steep increase in Google searches for specialized joint music-making platforms since mid-March (see Figure 1) suggests that, at least to some extent, musicians turned to virtual means of musical interaction.

Most virtual platforms designed to play music with others claim to enable musicians to jam in real time over the Internet (e.g., JamKazam, Jamulus, and JackTrip). However, online musicians’ forums and blogs reveal that latency issues, often prompted by distances between the users (and server), networking efficiency, and quality of the routing services, cause feelings of frustration, and often make it difficult for more than two musicians to actually play in synchrony (e.g., Dolister, 2020; Marraccini, 2020). The importance of synchronization in successful musical interaction, and its ability to foster feelings of social connectedness specifically, have been demonstrated in a number of previous studies (see, e.g., Hove and Risen, 2009; Marsh et al., 2009; Wiltermuth and Heath, 2009; Valdesolo and Desteno, 2011; Demos et al., 2012; Leman, 2016; Stupacher et al., 2017). Furthermore, latency and the disruption of synchronizing abilities might interfere with a successful sharing of intentionality in the musical interaction; a concept related to social behavior (Tomasello et al., 2005; Reddish et al., 2013; Harris and Küssner, 2020). As this shared intentionality (i.e., the coordination of action toward a common musical goal) can be communicated in milliseconds, any latency issue will inhibit successful musical interaction and related feelings of connectedness. Moreover, when considering online musical interaction, a notion receiving increased attention is that of presence. Presence relates to the subjective evaluation of the degree of “being there” and to the objective extent to which individuals behave in a digital environment, similar to the way they would do so in comparable everyday offline circumstances (Slater and Wilbur, 1997). Previous research indicated high graphics-update rate, low latency, and high degree of interactivity as some of the factors that contribute to a high sense of presence (Barfield and Hendrix, 1995), and discussed their importance for achieving feelings of togetherness in such environments (Durlach and Slater, 2000). It could be questioned to what extent platforms designed for real-time joint music making can actually facilitate the experience of togetherness equivalent to its experience instigated through live music interaction.

The aim of this study was to provide a scientific account describing the conditions of joint music making during the first weeks of lockdown, and assess to what extent social aspects were of importance herein. Through a survey, information was obtained on how and why musicians played music with others before and during lockdown, focusing on specifics of the setting (live or virtual), group composition, use and assessment of specific joint music-making platforms, and argumentation for playing with others. Additionally, respondents’ drive to make music with others, as well as their social and technical competence were assessed. We hypothesized that musicians would try to adapt to the lockdown measures through exploration of alternative methods for joint music making. Based on the clearly demonstrated shift of live music events to online platforms (e.g., Palamar and Acosta, 2020; Ren, 2020; Thomas, 2020; Weaver et al., 2020) and Google search results for joint music-making platforms, we expected musicians to mainly turn to virtual means of musical interaction with others, possibly preferring real-time methods, which more closely resemble live environments than non-real-time methods. However, we also hypothesized to observe reports of dissatisfaction with such real-time methods due to problems stemming from latency issues (i.e., decreasing levels of synchronization, shared intentionality, and presence). The obtained results could shed more light on the applied strategies of musicians to adapt to the early 2020s lockdown restrictions precipitated by the COVID-19 pandemic, as well as their assessment of these behavioral shifts. In addition, findings might provide valuable insights for musicians, teachers, conductors, music platform designers, as well as other stakeholders, potentially fueling future lockdown or quarantine settings.

## Materials and Methods

### Data Collection

The survey was administered in Microsoft Forms and distributed online from April 20 to May 1, 2020. Musicians were invited to participate through a range of online channels, mainly targeted at specific music communities (e.g., websites and social media channels of conservatories, peer-to-peer musician groups, universities, etc.). The survey was anonymous and all procedures were approved by the ethical committee of the authors’ institution. Respondents could opt to fill out the survey in Dutch or English and were informed that it would take approximately 20 min to complete. They received no financial compensation.

### Measures

The survey (see Supplementary Material) included multiple-choice questions, ranking questions, open-ended questions, and Likert scales (with incrementing ranks referring to increasing importance of the tested item, e.g., “1 = not important/not active/not pleasant” and “7 = extremely important/extremely active/extremely pleasant”). It consisted of the following three sections:

(1) *General information*: This contained questions on general demographics, as well as self-assessments of social and technical competence.
(2) *Joint music making before lockdown*: This section ascertained respondents’ musical behavior before lockdown (e.g., frequency and methods of playing with others). Three method categories were distinguished: (1) physically playing together, (2) use of (specialized) online real-time communication platforms (e.g., Zoom, JamKazam), and (3) use of alternative remote methods (e.g., recording musical parts separately and pasting them together at a later stage, playing with pre-recorded materials). If respondents were experienced with online real-time platforms, they were asked to assess their most successful experience using the following assessment criteria:
  (i) the ability to successfully play music with others in real time,
  (ii) the pleasantness of the experience,
  (iii) the effectiveness of the experience in reaching feelings of social connectedness,
  (iv) the similarity of the experience to playing physically together in a live setting,
  (v) the ability to reach an intended goal, and
  (vi) the ability to synchronize one’s performance with that of others.
Furthermore, we assessed general music-making behavior: intensity of active pursuit to play music with others, importance of social connectedness, motivation for joint music making (i.e., to have a good time, to feel connected to others, to improve musical skills, to maintain/expand networks, to express themselves creatively/personally, and to earn money), group composition, and lastly, to what extent they felt held back by their own technical capabilities.
(3) *Joint music making during lockdown*: Similar to the previous section, respondents’ musical behavior during lockdown was ascertained here. Additional questions were included regarding motivations for adjustments in playing frequency and shifts in urge to play. Respondents were also asked about the extent to which they missed playing music with others and whether they believed their musical network had changed.

In the survey, it was explicitly stated that all questions concerned joint music making that did not pertain to music education or therapy (with the exception of one item regarding musical activity as income source). Respondents were granted the opportunity to leave comments/remarks before submitting their responses.

### Data Analysis

Data were processed in Microsoft Excel. R version 4.0.2 (R Core Team, 2020) was used for data analysis. All functions used were part of the base R environment.

## Results

### Respondents

In total, 234 (123 females, 110 males; one preferred not to disclose) valid responses were collected. Ages ranged from 18 to 74 years (*M* = 38.501, *SD* = 13.053). The majority of the respondents resided in Belgium (81%), while 14% lived in the Netherlands. Both governments were enforcing social distancing regulations at the time, and the border between these countries was closed. The remaining 5% lived in a variety of countries, including Canada, Czechia, Denmark, France, Italy, the Philippines, and the United States. All mentioned countries were imposing some form of government-instructed lockdown.

Respondents indicated to have obtained or to currently pursue higher education (80%), secondary education (9%), post-university education (i.e., Ph.D.) (7%), or additional professional education (4%). As it was an inclusion criterion, all respondents (100%) were musicians experienced in joint performance. Years of (in)formal music training ranged from 2 to 50 years (*M* = 16.598, *SD* = 9.342); they received musical training at music schools (58%), through private lessons (30%) or self-education (30%), at conservatories (27%) or universities (5%) (e.g., musicology), or through a combination of the aforementioned. Respondents played a wide variety of instruments (see Table 1). Based on Schouten (2009), instruments were categorized as chordophones, aerophones, or percussion. Voice was added as an additional category. Of all respondents, 53% played chordophones, 53% aerophones, and 15% percussive instruments, while 34% indicated to sing. More than half of the respondents (55%) were multi-instrumentalists (including voice and multiple instruments within one category, e.g., guitar and ukulele). Of all respondents, 18% indicated to depend on their musical activities as a main source of income.

**TABLE 1:** Instruments played by respondents sorted by instrument category.

**Chordophones**		**Aerophones**	
*Bowed instruments*	*n*	*Woodwinds*	*n*
Cello	4	Bagpipes	1
Viola	1	Bassoon	2
Viola da gamba	1	Clarinet	28
Violin	10	Flute	28
		Oboe	3
*Plucked instruments*		Panflute	1
Banjo	4	Piccolo	3
Bass guitar	24	Recorder	6
Double bass	2	Saxophone	31
Guitar	60	Tin whistle	1
Ukulele	7	Traverso	1
*With keyboard*		*Brass*	
Piano/keyboard	80	Bugle	6
		Cornet	6
**Percussion**		Euphonium	7
*General*		Flugelhorn	1
Percussion/drums	32	Horn	7
		Trombone	7
*Membranophones*		Trumpet	12
Bodhran	1	Tuba	6
Drum (singular)	1		
		*With keyboard/free reed*	
*Idiophones*		Accordion	5
Cajon	1	Harmonica	1
Gamelan	1	Organ	3
Marimba	3		
Vibraphone	1	**Other**	
Xylophone	2	Robots	1
		DIY computer (software)	1
**Voice**	79	Synthesizer	2

### General Joint Music-Making Behavior

**FIGURE 2:**
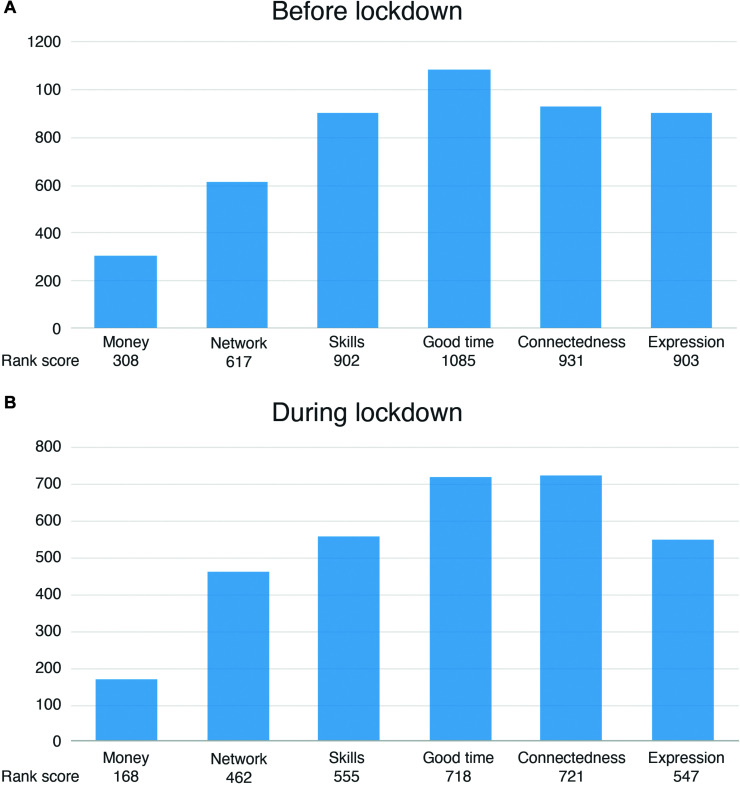
**(A)** Respondents’ ranked argumentations for playing music with others before lockdown. **(B)** Respondents’ ranked argumentations for playing music with others during lockdown. A higher score (provided per category on the *x*-axes) indicates a higher rank. The provided categories refer to: earning money (Money), maintaining/expanding networks (Network), improving musical skills (Skills), having a good time (Good time), feeling connected to others (Connectedness), and expressing oneself creatively/personally (Expression). A portion of respondents did not continue to make music with others in any form during lockdown, resulting in lower overall scores. While having a good time was indicated to be the most important reason to make music before lockdown, experiencing social connectedness gained importance during lockdown.

Assessment of the relevance of social connectedness in joint music making before lockdown indicated that 7% (*n* = 17) of all respondents did not regard social connectedness as important (scoring 1–3), while 88% (*n* = 205) considered it as a relevant factor (scoring 5–7). A large group of respondents (49%; *n* = 114) indicated to regard it as extremely important (scoring 7). During lockdown, 22% (*n* = 51) deemed social connectedness as rather unimportant (scoring 1–3), while 61% (*n* = 144) regarded it as a crucial factor (scoring 5–7). A paired samples Wilcoxon rank sum test demonstrated that social connectedness was regarded as significantly more important before lockdown (*Mdn* = 6) than during (*Mdn* = 5), *V* = 9832.5, *p* < 0.001, *r* = 0.375. Moreover, Figure 2 displays ranked motivators for joint music making. Scores were calculated based on the given rank, with higher scores relating to higher ranks [*n*^∗^1 (rank 6) + *n*^∗^2 (rank 5)… + *n*^∗^6 (rank 1)]. Results indicated that, while overall enjoyment was regarded as fundamental before as well as during lockdown, the importance of social connectedness increased during lockdown.

Furthermore, 9% (*n* = 22) did not actively pursue joint music making before lockdown (scoring 1–3), while 85% (*n* = 200) reported to have actively done so (scoring 5–7). During lockdown, 57% (*n* = 133) of the respondents proved to be rather inactive (scoring 1–3), whereas 29% (*n* = 67) did report to have actively pursued playing with others (scoring 5–7). A paired samples Wilcoxon rank sum test showed higher levels of activity before (*Mdn* = 6) than during lockdown (*Mdn* = 3), *V* = 20476, *p* < 0.001, *r* = 0.522. Closer examination of urge to play with others provided more context to these findings; 16% (*n* = 38) indicated a decrease in urge to play during lockdown (scoring 1–3), while 41% (*n* = 95) reported an increase (scoring 5–7). Of those signaling a decreased urge, 50% (*n* = 19) provided argumentation relating to social aspects (e.g., “lack of human contact,” “loss of social connection,” “missing personal interaction”). Those reporting an increase related this to a need to feel socially connected to others (89%; *n* = 85), to cope with the situation and stress (63%; *n* = 60), and to aid in tackling boredom (45%; *n* = 43).

Moreover, 8% (*n* = 19) reported not to miss playing with others during compared with before lockdown (scoring 1–3), whereas 84% (*n* = 196) did (scoring 5–7). The majority of respondents (56%; *n* = 131) indicated to miss it extremely (scoring 7). Spearman rank correlation analyses showed that the degree to which individuals missed joint music making was positively correlated with their level of active pursuit to play with others, *r*_*s*_ = 0.228, *p* < 0.001, and their appraisal of social connectedness in joint music making during lockdown, *r*_*s*_ = 0.455, *p* < 0.001, as well as their self-assessed level of social competence, *r*_*s*_ = 0.165, *p* = 0.012.

Self-assessment of social competence level ranged between 2 and 7 (*Mdn* = 5), with Wilcoxon rank sum test demonstrating females (*Mdn* = 6) to have assessed their level as significantly higher than males (*Mdn* = 5), *W* = 8162, *p* = 0.005, *r* = 0.183. Levels of self-assessed technical competence varied between 1 and 7 (*Mdn* = 5), with male respondents (*Mdn* = 5) scoring significantly higher than females (*Mdn* = 4), *W* = 4705, *p* < 0.001, *r* = 0.268.

Lastly, when compared with before lockdown, an overall decrease of 53% was observed in music group (e.g., choir, band, orchestra) participation during lockdown (see Table 2). Although less engagement was demonstrated for most composition types, an increase was revealed for interaction with co-habitants. When regarding the size of their musical network, 60% (*n* = 141) signaled decreases (scoring 1–3), while only 7% (*n* = 17) reported increases in network size (scoring 5–7).

**TABLE 2:** Group compositions before and during lockdown.

	Group compositions	*n*
**Before lockdown (*N* = 234)**	Choir/singing group	53
	Band/jam	90
	Orchestra/harmony/ensemble/brass	149
	Cohabitants	2
**During lockdown (*N* = 228)**	Choir/singing group	23
	Band/jam	43
	Orchestra/harmony/ensemble/brass	53
	Cohabitants	17

### Joint Music Making: Physically Present

Before lockdown, all respondents (*N* = 234) played music with others. Yet, during lockdown, this number decreased to 21% (*n* = 49) (see Table 3a). Next to playing occurrence, a Wilcoxon rank sum test demonstrated significantly lower playing frequencies during (*Mdn* = 2 or “once a week”) compared with before lockdown (*Mdn* = 1 or “more than once a week”), *W* = 8100, *p* < 0.001, *r* = 0.299. Spearman analyses demonstrated a significant correlation of playing frequency before lockdown with social self-assessment, *r*_*s*_ = −0.147, *p* = 0.024. In addition, Kruskal–Wallis tests revealed higher playing frequencies before lockdown for those who were dependent on musical activities as a main income source (*Mdn* = 1 or “more than once a week,” *M* = 1.390, *SD* = 0.862) compared with respondents depending on other revenue streams (*Mdn* = 1 or “more than once a week,” *M* = 1.635, *SD* = 0.906), *H*(1) = 5.084, *p* = 0.024, *r* = 0.147 (see Table 3b).

**TABLE 3:** Joint music-making behavior before and during lockdown: physically present.

3a.	*Playing occurrence*				
	
	Yes	No			
Before	234 (100%)	0 (0%)			
During	49 (21%)	185 (79%)			

	***Playing frequency***				
	
	**>Once a week**	**Once a week**	**Once every 2 weeks**	**Once a month**	**<Once a month**

Before	138 (59%)	73 (31%)	8 (3%)	9 (4%)	6 (3%)
During	14 (6%)	15 (6%)	6 (3%)	1 (<1%)	13 (6%)

**3b.**	***Interaction effects of playing occurrence and frequency before lockdown***				
	
		Playing occurrence		Playing frequency	
Variables		*Using Wilcoxon (W)*		*Using Kruskal-Wallis (H)*	

Household type		–		8.738 (*p* = 0.120)	
Employment situation		–		7.56 (*p* = 0.056)	
Music as main income		–		5.084 (*p* = 0.038)*	
		–		*Using Spearman (r_*s*_)*	
				
Technical competence				−0.032 (*p* = 0.628)	
Social competence		–		−0.147 (*p* = 0.024)*	

**3c.**	***Interaction effects of playing occurrence and frequency during lockdown***				
	
		Playing occurrence		Playing frequency	
Variables		*Using Wilcoxon (W)*		*Using Kruskal-Wallis (H)*	

Household type		4425 (*p* = 0.792)		5.883 (*p* = 0.208)	
Employment situation		4835 (*p* = 0.444)		1.442 (*p* = 0.696)	
Music as main income		4462.5 (*p* = 0.809)		0.948 (*p* = 0.330)	
				*Using Spearman (r_*s*_)*	
				
Technical competence		4665.5 (*p* = 0.748)		−0.036 (*p* = 0.804)	
Social competence		4748 (*p* = 0.600)		−0.141 (*p* = 0.332)	

(3a) Playing occurrence indicates whether respondents played music with others (Yes) or not (No) before and during lockdown. Playing frequency indicates how often respondents played. (3b and 3c) Summarized results of Wilcoxon rank sum and Kruskal-Wallis tests on variables with possible influence on musical behavior are provided, as well as results of Spearman corrleation tests on self-assessed technical and social competence (*significant effect). Playing occurrence before lockdown was 100%, so no comparison could be made here between those who did and did not play with others.

Interaction tests between household, employment, main income source, and competence variables on the one hand, and playing occurrence and frequency during lockdown on the other, unveiled no significant effects (see Table 3c). Individuals who played physically together with others during lockdown indicated that this occurred in the same room (76%; *n* = 37), in the street (e.g., with neighbors) (12%; *n* = 6), or in both these situations (12%; *n* = 6).

### Joint Music Making: Online Real-Time Methods

Of all respondents, 5% (*n* = 11) indicated to have obtained previous experience with online real-time joint music-making methods. During lockdown, experience levels increased with 264%, as 17% (*n* = 40) indicated to have used such methods. In addition, the usage frequency of such methods significantly increased during lockdown (*Mdn* = 2 or “once a week”), when compared with before (*Mdn* = 5 or “less than once a month”), *W* = 3640, *p* < 0.001, *r* = 0.472 (see Table 4a). Increased playing frequencies were uncovered for those depending on music as a main income source (*Mdn* = 1, or “more than once a week”) compared with individuals depending on other income means (*Mdn* = 2.5, or “about once a week”/”once every 2 weeks”), *H*(1) = 7.498, *p* = 0.006, *r* = 0.433 (see Table 4b).

**TABLE 4:** Joint music-making behavior before and during lockdown: online real-time methods.

4a.	*Playing occurrence*				
	
	Yes	No			
Before	11 (5%)	221 (95%)			
During	40 (17%)	194 (83%)			

	***Playing frequency***				
	
	**>Once a week**	**Once a week**	**Once every 2 weeks**	**Once a month**	**<Once a month**

Before	0 (0%)	1 (<1%)	0 (0%)	4 (2%)	6 (3%)
During	9 (4%)	14 (6%)	6 (3%)	5 (2%)	6 (3%)

**4b.**	***Interaction effects of playing occurrence and frequency during lockdown***				
	
		Playing occurrence		Playing frequency	
Variables		*Using Wilcoxon (W)*		*Using Kruskal-Wallis (H)*	

Household type		3711.5 (*p* = 0.654)		4.708 (*p* = 0.319)	
Employment situation		3272.5 (*p* = 0.097)		2.425 (*p* = 0.489)	
Music as main income		4090 (*p* = 0.432)		7.498 (*p* = 0.006)*	
				*Using Spearman (r_*s*_)*	
				
Technical difficulty		3894 (*p* = 0.930)		0.179 (*p* = 0.268)	
Technical competence		4095 (*p* = 0.575)		−0.001 (*p* = 0.993)	
Social competence		4103.5 (*p* = 0.558)		−0.051 (*p* = 0.755)	

(4a) Playing occurrence indicates whether respondents played music with others using online real-time methods (Yes) or not (No) before and during lockdown. Playing frequency indicates how often respondents played. (4b) Summarized results of Wilcoxon rank sum and Kruskal-Wallis tests on variables with possible influence on musical behavior are provided, as well as results of Spearman correlation tests on experienced technical difficulty, self-assessed technical and social competence (*significant effect).

Overall, the following platforms were employed: Zoom (*n* = 18), Skype (*n* = 17), Messenger (*n* = 14), Microsoft Teams (*n* = 7), Google Hangouts (*n* = 6), WhatsApp (*n* = 5), Jitsi (*n* = 3), Facetime (*n* = 2), Facebook Live (*n* = 2), Instagram Live (*n* = 1), JackTrip (*n* = 1), JamKazam (*n* = 1), JamTaba (*n* = 1), Jamulus (*n* = 1), Ninjam (*n* = 1), SoundJack (*n* = 1), Starleaf (*n* = 1), and Whereby (*n* = 1). Additionally, some tailored approaches were used (e.g., combinations of Max, SuperCollider, Cubase, and/or Pure Data). Online platforms specifically designed to play music with others included JackTrip, SoundJack, JamTaba, Ninjam, JamKazam, and Jamulus (note: JamTaba and Ninjam do not allow for real-time interaction as time delay is added in order to synchronize the timing. This allows musicians to synchronize their performance with sets of bars, but, although the performance will be perceived as in time, the actual input/output is delayed).

#### Assessment

Interestingly, 23% (*n* = 9) of the respondents who used these platforms during lockdown indicated that none of them yielded successful experiences, with 13% (*n* = 5) signaling none of the commercially available platforms to be suitable for joint music making. Although another 13% (*n* = 5) did provide positive comments regarding some of the platforms (e.g., quality of sound), this portion of the sample indicated not to have used them simultaneously with other musicians (i.e., they took turns in playing). Furthermore, some platforms allocated to the most successful experience category also appeared in the least successful one, usually due to latency issues (see Table 5).

**TABLE 5:** Commentary on most and least successful experiences with online real-time methods.

Most successful	Comments	Freq.	Least successful	Comments	Freq.
	None of the employed platforms was succesful	9		All of the employed platforms were unsuccesfull	5
Zoom	Good (sound) quality (*n* = 3); not used simultaneously; latency issues	6	Zoom	Latency (*n* = 4); cannot be used simultaneously	7
Facetime	Good sound quality; good for giving lessons	3	–		
Personal set-up		2	–		
Messenger	Not used simultaneously	2	Messenger	Latency (*n* = 2); cannot be used simultaneously; bad sound quality	3
WhatsApp	Not used simultaneously; good sound quality	2	WhatsApp	Latency; bad sound quality; connectivity problems	3
JamKazam		1	–		
Skype	User friendly	1	Skype	Latency (*n* = 4); bad sound quality; connectivity problems	8
Jitsi	Not used simultaneously	1	Jitsi	Cannot be used simultaneously	3
JamTaba	Not used simultaneously	1	JamTaba	Not in real time	1
Google Hangouts	However, sound issues	1	Google Hangouts		1
Microsoft Teams		1	Microsoft Teams	Latency of video; not user friendly	4
Jamulus	Easy to use; latency ok	1	–		
Whereby	Easy to use	1	–		

*Summary of provided commentary on respondents’ experiences with the least and most successful platform in mind.*

**FIGURE 3:**
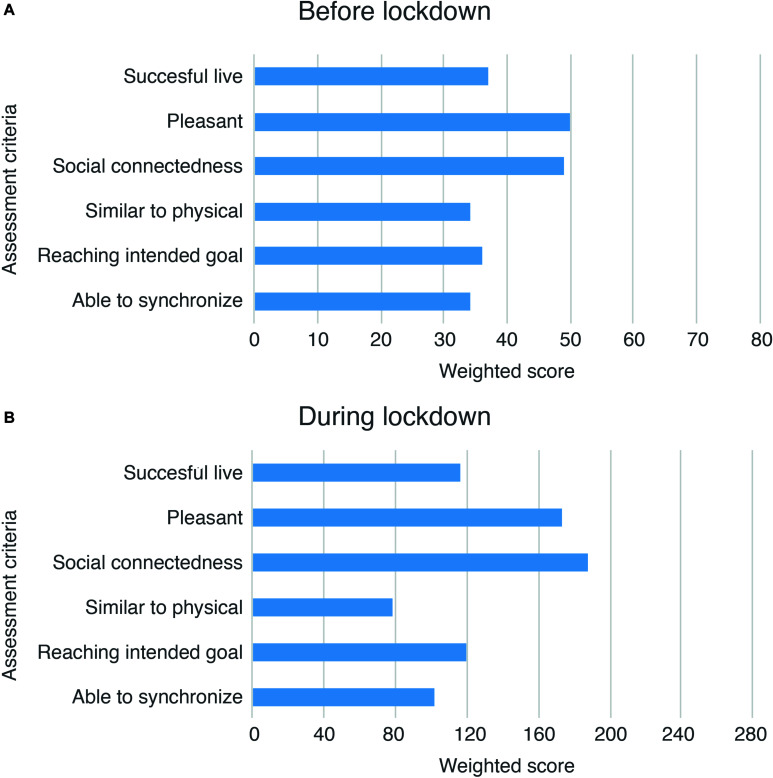
**(A)** Weighted scores on six criteria used for assessment of user experience of online real-time methods before lockdown. **(B)** Weighted scores on six criteria used for assessment of user experience of online real-time methods during lockdown. A higher score indicates a more satisfactory experiences. Scores were calculated by multiplying the frequency of a given answer with the corresponding Likert score (*n*^∗^1 + *n*^∗^2 … + *n*^∗^7), resulting in a maximum score of 77 before lockdown and 280 during lockdown, as more respondents used online real-time methods during than before lockdown. Assessment criteria refer to: the ability to successfully play music with others in real time (Succesful live), the pleasantness of the experience (Pleasant), the effectiveness of the experience in reaching feelings of social connectedness (Social connectedness), the similarity of the experience to playing physically together in a live setting (Similar to physical), the ability to reach an intended goal (Reaching intended goal), and the ability to synchronize one’s performance with that of others (Able to synchronize).

Respondents’ assessments of their most successful platform experience demonstrated that, except for pleasantness and the ability to feel a social connection, most criteria scored below average (see Figure 3).

A small portion of the respondents (*n* = 3) positively assessed JamKazam, Jamulus, JackTrip, and SoundJack regarding the proficiency of these platforms to enable live joint music making, generate pleasant experiences, provoke feelings of social connectedness, facilitate intended goal achievement, and enable synchronization (scoring 5–7). However, respondents stressed that the experiences created through the use of these platforms did not equal those established through common offline musical interaction.

#### Lack of Utilization

Respondents who refrained from using online real-time methods during lockdown (*n* = 194) provided the following arguments for doing so; 42% (*n* = 82) indicated not to feel the need to try such methods; 24% (*n* = 46) stated they knew from personal experience, and 20% (*n* = 38) from experiences of others, that such methods would not work (for them); 14% (*n* = 28) expressed not to have been aware of the existence of such methods; 10% (*n* = 20) said they lacked the time to use them; 2% (*n* = 4) provided technical arguments (e.g., lacking equipment or technical skills).

### Joint Music Making: Alternative Remote Methods

Before lockdown, 29% (*n* = 67) of all respondents played music with others using alternative remote methods. During lockdown, this increased to 55% (*n* = 129; an increase of 93%) (see Table 6a). Overall, no significant interaction effects were retrieved for playing occurrence or frequency and variables concerning household type, employment situation, income source, or technical and social competence (see Table 6b). Of all respondents who used alternative methods during lockdown, 78% (*n* = 101) reported to have recorded their parts separately and subsequently pasted these parts digitally, 2% (*n* = 3) indicated to have played along with pre-recorded material (e.g., using Spotify, YouTube), while 18% (*n* = 23) made use of both aforementioned methods.

**TABLE 6:** Joint music-making behavior before and during lockdown: alternative remote methods.

6a.	*Playing occurrence*				
	
	Yes	No			
Before	67 (29%)	161 (71%)			
During	129 (55%)	105 (45%)			

	***Playing frequency***				
	
	**>Once a week**	**Once a week**	**Once every 2 weeks**	**Once a month**	**<Once a month**

Before	–	–	–	–	–
During	26 (11%)	30 (13%)	30 (13%)	25 (11%)	18 (8%)

**6b.**	***Interaction effects of playing occurrence and frequency during lockdown***				
	
		Playing occurrence		Playing frequency	
Variables		*Using Wilcoxon (W)*		*Using Kruskal-Wallis (H)*	

Household type		6889 (*p* = 0.815)		3.591 (*p* = 0.609)	
Employment situation		6793.5 (*p* = 0.966)		5.675 (*p* = 0.129)	
Music as main income		6640 (*p* = 0.708)		1.808 (*p* = 0.405)	
				*Using Spearman (r_*s*_)*	
				
Technical difficulty		6265.5 (*p* = 0.317)		−0.030 (*p* = 0.733)	
Technical competence		7725.5 (*p* = 0.059)		0.034 (*p* = 0.704)	
Social competence		6731 (*p* = 0.935)		−0.092 (*p* = 0.302)	

*(6a) Playing occurrence indicates whether respondents played music with others using alternative remote methods (Yes) or not (No) before and during lockdown. Playing frequency indicates how often respondents played. (6b) Summarized results of Wilcoxon rank sum and Kruskal-Wallis tests on variables with possible influence on musical behavior are provided, as well as results of Spearman correlation tests on experienced technical difficulty, self-assessed technical and social competence.*

#### Assessment

When elaborating on the most successful experience using alternative methods, 66% (*n* = 85) indicated the recording of separate parts and subsequent circulation of these materials as the most favored alternative method, while only 5% (*n* = 6) reported to prefer playing along with pre-recorded tracks. With regard to the first approach, 19% (*n* = 16) preferred it due to its lack of related latency issues (as compared with online real-time methods), while 15% (*n* = 13) provided (partly) negative comments relating to the lack of eye contact, the inability to read body language and observe feet tapping, etc.

**FIGURE 4:**
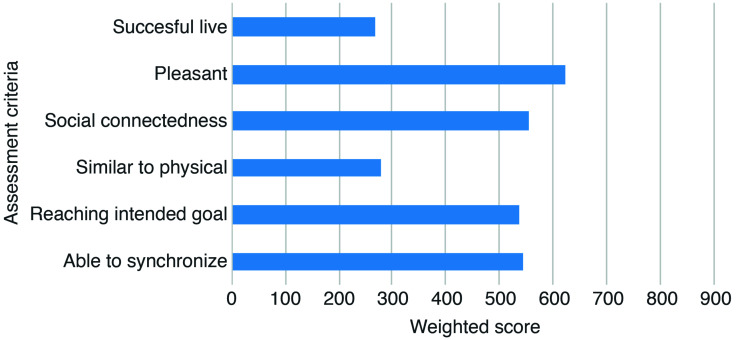
Weighted scores on the six criteria used for assessment of user experience are shown of alternative remote methods, with a higher score indicating more satisfactory experiences. Scores were calculated by multiplying the frequency of a given answer with the corresponding Likert score (*n*^∗^1 + *n*^∗^2 + … + *n*^∗^7), resulting in a maximum score of 903.

Additionally, ratings of assessment criteria of alternative remote methods demonstrated rather low overall ratings. However, pleasantness and the ability to feel a social connection, to reach an intended goal, and to synchronize one’s playing with that of others scored above average (see Figure 4).

Spearman correlation analyses revealed negative associations between experienced technical difficulty and assessed pleasantness, *r*_*s*_ = −0.252, *p* = 0.004, ability to experience social connectedness, *r*_*s*_ = −0.224, *p* = 0.011, to reach an intended goal, *r*_*s*_ = −0.216, *p* = 0.014, and to synchronize musical output, *r*_*s*_ = −0.307, *p* < 0.001. This indicates that respondents who experienced more technical difficulties assessed the employed methods more negatively on these criteria. For the other two criteria, no significant correlations with experienced technical difficulty were found (i.e., ability to successfully play live, *r*_*s*_ = 0.037, *p* = 0.678; similarity to playing while being physically together, *r*_*s*_ = −0.136, *p* = 0.131).

#### Lack of Utilization

Those who refrained from alternative remote methods’ use (*n* = 105) explained it as such: 47% (*n* = 60) reported not to feel a need to try them; 18% (*n* = 23) said they lacked the time to try these methods, 14% (*n* = 18) argued they knew from personal experience, and 15% (*n* = 19) from experiences of others, that it would not work (for them), while 10% (*n* = 13) expressed not to have been aware of the existence of such methods.

### Comparison of Joint Music-Making Methods

Wilcoxon rank sum tests demonstrated significant differences between online real-time and alternative methods for three of the six assessment criteria. Alternative remote methods (*Mdn* = 1) were shown to score lower than online real-time methods (*Mdn* = 2) regarding their proficiency to enable live joint music making, *W* = 1944, *p* = 0.009, *r* = 0.201. On the other hand, alternative methods (*Mdn* = 4) were assessed more positively than online real-time ones (*Mdn* = 3) with respect to their ability to facilitate intended goal achievement, *W* = 3442.5, *p* = 0.001, *r* = 0.248, and additionally scored higher (*Mdn* = 4) than online real-time methods (*Mdn* = 2) regarding their proficiency to enable synchronization, *W* = 3743, *p* ≤ 0.001, *r* = 0.335.

## Discussion

The goal of this study was to provide a scientific account describing the conditions of joint music making during the first weeks of lockdown induced by the COVID-19 pandemic of 2020 compared with the situation before. Information is provided regarding playing occurrence and frequency, as well as employed methods. In addition, user experiences of the used methods and motivators of joint music making are described, with a specific focus on experiences of social connectedness.

### Landscape of Joint Music Making During the COVID-19 Pandemic

Frequency of joint music making in live music settings decreased substantially during lockdown when compared with the pre-lockdown situation. This finding is rather straightforward, as respondents were officially not allowed to interact with more than one other individual while being physically present, and such gatherings could only take place outdoors. Some respondents did indicate to have moved their live jams/rehearsals to outdoor environments (e.g., streets), as such behaving in compliance with governmental restrictions, while others played with fellow musicians in the same room. Although an increase in joint music making with co-habitants was observed, this specific population alone could not account for this effect; possibly, these results signal some violation of the imposed regulations. A decrease was also found in variety of configurations respondents played in. Moreover, most stated that their musical network had decreased during lockdown. Again, these results might seem rather evident, but if we consider that one of the most often-mentioned appeals of online joint music-making platforms is their ability to increase accessibility by providing the opportunity to play with anyone anywhere (Iorwerth and Knox, 2019), we could have assumed such platforms to preserve (or even increase) group composition variety and network size. Yet, only 7% of respondents indicated a (slight) increase, of whom only two specifically indicated that online methods indeed increased joint music-making opportunities through improved accessibility.

While substantial increases in online platform use were observed during lockdown, the overall portion of respondents using such platforms remained rather low (17%). Our data suggest that this hesitant behavior might be explained by a rather negative overall stance toward these platforms, as 44% of the respondents claimed that such tools “would not work for them.” Remarkably, only a small number (7%) of platforms employed during lockdown were specifically designed for online joint music making (e.g., JamKazam, Jamulus), with the lion’s share (93%) consisting of common video-calling and conferencing tools (e.g., Zoom, Skype). Thus, even though our respondents significantly increased their use of online real-time platforms to jam and/or rehearse with others during lockdown, they mostly depended on tools they were already acquainted with and/or heard from by others, rather than exploring those specifically targeted at online joint music making.

To a certain extent, this could be explained by the fact that some respondents were unaware of the existence of such tools. Alternatively, the steep learning curve to operate these specialized platforms, with a general need for technical know-how, might partly explain the tentative attitude of our respondents as well. Previous research has suggested that people are primarily incentivized to engage, or persevere, in activities when they expect to be successful in them and/or do not envision significant difficulties (Bandura, 2009). Thus, beliefs of self-efficacy might have played a role here. Correspondingly, the need for specific equipment that can enable the creation of satisfactory setups (e.g., audio interface, direct internet connection), might have played a role as well. Indeed, some provided statements such as “I do not have the right equipment,” or “We are experiencing too many technical barriers.” Additionally, they might have regarded the endeavor to scout for specific platforms, as well as learn how to work with them, as more time-consuming (and/or having a more unknown outcome) than simply relying on video-conferencing/calling tools they were already acquainted with.

Furthermore, the generally negative assessment of online platforms could provide some explanation to only 17% of respondents using such methods. As hypothesized, such assessments mostly related to their inefficiency to enable synchronization, facilitate intended goal achievement, and resemble real-life joint music-making experiences. Latency issues were often explicitly mentioned as a distorting factor. To overcome such issues, some respondents took turns while playing, rather than playing simultaneously. Some respondents specifically stated that none of the used platforms were suitable for professional music making.

Although scores of overall capability of platform use to facilitate feelings of pleasantness and social connectedness were above average, scores of aptitude to support synchronization were below average. This is rather surprising since previous research proposed a link between synchronization and experienced feelings of social connectedness (e.g., Hove and Risen, 2009; Marsh et al., 2009; Wiltermuth and Heath, 2009; Valdesolo and Desteno, 2011; Demos et al., 2012; Leman, 2016; Stupacher et al., 2017). To some extent, higher appraisals of pleasantness and social connectedness might be explained by the unprecedented context of social deprivation prompted by the lockdown, during which any means of (musical) interaction, albeit unsatisfactory, could have promoted social connectedness simply because it provided a shared experience. Such a rationale is well in line with research stressing the facilitation of overall positive mood states through most forms of musical interaction (La Lamont, 2012; Croom, 2015).

Our results further demonstrated an impact of income source; musicians indicating to financially gain from musical activities were demonstrated to turn more swiftly to such platforms, suggesting a more flexible attitude and/or adaptation strategy of this subgroup. This might be due to financial gain acting as an extra incentive, as such serving as an additional facilitator of behavioral adaptation, whereas a lack of financial benefit implied less enticement. Interestingly, in contrast to other recent studies (Ribeiro et al., 2021; Spiro et al., 2021), this was the only demographic indicator that resulted in a significant interaction with musical behavior during lockdown.

Next to online real-time methods, a large increase in use of alternative methods for remote joint music making during lockdown was seen as well (mainly recording separate parts and subsequent circulation of these materials). While this increase was less dramatic than the boost in online platform use, more than half of respondents (55%) continued to play music using such alternative methods. Superior appraisals of alternative methods concerning goal achievement and synchronization capabilities could be owing to the fact that respondents did not play together in real time when using alternative methods, thus canceling out latency issues. This is consistent with statements of respondents who indicated to have turned to alternative methods in order to circumnavigate latency issues. However, user accounts revealed negative aspects as well, with some referring to a lack of essential, subjectively perceived features of joint music making (e.g., eye contact, the capacity to read each other’s body language, the ability to feel the energy in the room). One of the respondents stated: “It is just not the same as being in a room with people and FEELING THE VIBRATIONS, smelling the sweat, seeing tapping feet (…) I need people in the room with me” (capitalization by respondent). Previous research stressed the role of such components as critical elements for communicating affective information in musical interaction (Vines et al., 2011; Vuoskoski et al., 2014, 2016).

A more positive assessment of online platforms regarding their facilitation of live (i.e., real-time) joint musical interaction in comparison with alternative methods could have been expected, as the latter does not enable real-time interaction. Alternative methods were, however, regarded as more effective means to reach intended goals and synchronize performances. Possibly, respondents had clearer predictions of what to expect using such methods and adjusted their intentions accordingly.

### General Motivations and Perceptions: The Role of Social Connectedness

Observed decreases of respondents’ efforts to musically interact with others during lockdown might to some extent be due to impaired circumstances to musically interact–since lockdown regulations firmly restricted live interactions between individuals–as well as to the overall negative view on available alternatives. Interestingly, this decreased effort was linked to the extent to which respondents reported to miss playing with others, which might signal an overall awareness of the negative corroborations of this diminished undertaking. As the feeling of missing to play with others correlated with self-assessed social competence and appraisal of social connectedness, this experience of loss might to some extent be due to social facets of musical interaction. This corresponds with reports of increased urges to musically interact with others (89%), as such referring to the facilitating ability of musical interaction to feel socially connected to others.

The relevance of social connectedness was further substantiated by findings on motivators of joint music making. Regardless of the employed tools, general enjoyment was shown to be the main reason to play with others before lockdown. During lockdown, social connectedness was indicated as key motivator. Yet, when testing the importance of social connectedness during music-making activities, a significant decrease was retrieved. Although these results seem to contradict at first glance, they should be interpreted in reference to the nature of questioning. When inquiring about arguments for joint music making, social connectedness turned out to be pivotal. In actual musical interactions, however, this item bared less relevance, since digitally evoked social contexts were generally considered as reduced settings (e.g., lacking contextual cues such as those experienced in real-life environments). As such, these findings suggest an increased “need,” combined with a decreased “experience,” of social connectedness during lockdown.

Our findings are well in line with the widely accepted notion that music is in essence a social activity (Nettl, 1983, 2000; Roederer, 1984; McNeill, 1995; Freeman, 2000; Dunbar, 2004). The overall yearning expressed by respondents to keep playing with others seems to be mainly driven by social aspects. Therefore, the generally perceived inability of digital methods to provide an adequate substitute for music making in live settings has meaningful implications. The deprivation of such musical interactions during a time of social distancing further paints a picture of the inadequacy to saturate social needs. It has been suggested that feelings of relatedness to others are one of three basic psychological needs (besides autonomy and competence, see Deci and Ryan, 2000), and research has shown that social isolation can have harmful physical and psychological health effects (House et al., 1988; Hawryluck et al., 2004; Barbisch et al., 2015; Brooks et al., 2020). Moreover, in line with other investigations (e.g., Durlach and Slater, 2000; Onderdijk et al., 2021), other aspects related to social connectedness, such as feeling present with others similarly as to live situations, also proved to be ineffectively facilitated by virtual music-making methods. Thus, our findings raise critical concerns regarding the future of virtual means of joint music making for possible forthcoming lockdown situations, as well as regarding their ability to adapt to a highly digitized world where shifts to the virtual realm are prevalent.

### Limitations and Future Directions

While this study provides an account of some of the strategies applied by musicians to deal with lockdown restrictions, no details of specific setups used for online music making were included. Although outside the scope of this paper, more in-depth investigation on the matter might have facilitated the formulation of more detailed recommendations for tool improvement. In addition, when administering the survey, we anticipated greater response from individuals with specialized music platform experience. The fact that the actual study sample did not meet this expectation could be regarded as a finding on its own, yet it also inhibited us to provide more general conclusions regarding this specific group.

Furthermore, multimodal functions varied between different platforms and were thus not controlled for. Jamulus, for instance, did not enable video recording at the time of surveying, while respondents could have opted to disable their camera (e.g., to preserve bandwidth) when using other platforms. Future research on the topic could employ better control mechanisms, for instance by regulating camera use. However, these functions could also be exploited in prospective inquiries. Techniques such as eye tracking could be applied to investigate the role of (attention to) visual information with respect to online musical interaction. Similarly, camera footage could be used to examine the level of synchronization in a more quantitative manner. Moreover, virtual reality (VR) settings could be explored, as VR was shown to yield promising results with regard to multimodal aspects of joint music making (Loveridge, 2020).

A wide variety of instruments was included in this study, as our aim was to report on a broad population (i.e., musicians) rather than focusing on a specific group of instrumentalists. Although some have explored aspects regarding the suitability of specific instruments or instrument types for online platform use (e.g., Davies, 2015), this field of research could benefit from further investigation, potentially also exploring specialized setups. Similarly, while plenty of musical genres have been defined, the current study focused on joint music making regardless of this aspect, although explorations regarding genre might be of interest as well. Particular musical genres might, for example, relate differently to timing, and could be less prone to negative experiences due to latency issues.

## Conclusion

To conclude, this study provided a scientific account of joint music making during the first weeks of lockdown induced by the COVID-19 pandemic of 2020 and compared it with the situation before lockdown. To our knowledge, such results were not reported before. Insights into the behavior of musicians as well as their motivations and perceptions to engage in or refrain from joint music making were presented, challenging the notion of online joint music-making tools as exhaustive substitutes for live music making. Often, lack of expertise and/or experience with digital joint music-making tools was observed. Such findings contrast with (digital) evolutions in music performance (e.g., livestreams, online jams) and education (e.g., online teaching, blended learning) and disclose a need for more adequate music training strategies (i.e., focusing on digital technologies and related technical skills) in order for musicians to keep up with a highly digitized world.

## Data Availability Statement

The raw data supporting the conclusions of this article will be made available upon request.

## Ethics Statement

The studies involving human participants were reviewed and approved by Ethics Commission Faculty of Arts and Philosophy Ghent University. The participants provided their written informed consent to participate in this study.

## Author Contributions

KO and EVD designed and executed the study. KO and FA performed the statistical analysis. KO and EVD prepared the manuscript. All authors read and approved the final manuscript.

## Conflict of Interest

The authors declare that the research was conducted in the absence of any commercial or financial relationships that could be construed as a potential conflict of interest.
