# Supplementary material for: Impact of Lockdown Measures on Joint Music Making: Playing Online and Physically Together
Source: Front Psychol. 2021 May 28;12:642713. doi: 10.3389/fpsyg.2021.642713 (PMC8193044; doi:10.3389/fpsyg.2021.642713)
Supplement: Supplementary file 1 [file Data_Sheet_1.docx]

Supplementary Material

# Supplementary Data

# Supplementary data includes the survey that was used to collect data. Both the English version and Dutch version are provided.

# To clarify, when bullet points are presented as ο, it means one answer could be selected. When bullet points were presented as □, multiple answers could be selected. “Other…” answers provided an opportunity for respondents to personalize their answer. Supplementary Material 1: Administered survey in English

**Making music together: offline and online**

Thank you for your willingness to participate in our study.

As researchers in musicology at Ghent University we are interested in how people make music with others. This concerns the period before and/or during the current lockdown period, particularly focusing on the use of existing online platforms.

In this survey we will ask you some questions on your personal/musical background and your musical behaviour both before and during the lockdown. Participation will take 20 minutes max.

Your anonymity and confidentiality will be maintained during the collection, processing, preserving, and reporting of collected data.

Please contact us if you have any questions/remarks.

Contact:

Kelsey Onderdijk (Kelsey.Onderdijk@ugent.be)

**Section 1**

**Informed Consent**

If you decide to participate in our study, we kindly ask you to state your approval regarding the following:

I declare that I wish to participate in a survey from Ghent University, and ...

(1) I have read the information on the nature of the experiment, the data that will be gathered, and I got the opportunity to ask additional questions (contact info on previous and last page)

(2) I am participating completely voluntarily in this study

(3) I give permission to process, preserve, and report my results anonymously

(4) I am aware that I can discontinue my participation at any time

(5) I realize that not participating or discontinuing to participate will in no way influence my evaluation and/or any tutoring I receive from Ghent University

(6) I can get a summary of the research findings, on request (contact info on previous and last page)

1. Have you read the above statements?

- Yes, I hereby consent to participate in this study according to the statements provided above.

**Section 2**

**Background information**

*Please answer in English or Dutch.*

2. What is your age?

3. What is your sex?

- Female
- Male
- X
- No answer
- Other…

4. What is your current country of residence?

5. In what type of household do you live?

- Living alone
- Couple, without kid(s)
- Couple, with kid(s)
- Living alone, with kid(s)
- Living with parent(s), family, friends or acquaintances
- Other…

6. What is your highest obtained degree? If currently a student, choose the degree you are currently pursuing.

- Primary education
- Secondary education
- Professional additional education (MBO/7th year/...)
- Higher education (University/University College/...)
- Post-university (PhD)
- Other…

7. What is your current work situation?

- Working full-time
- Working less than full-time
- Unemployed (temporary or other)
- Student
- Other…

8. Is your personal main source of income dependent on your musical activities (teaching included)?

- Yes
- No
- Other…

9. Which type of formal/informal music training did you receive/are you currently receiving?

- Music school
- Private lessons
- Self-instruction (autodidact)
- Conservatory
- Musicology
- I never received any music training
- Other…

10. Which instrument(s) (voice included) do you currently play?

11. In total, how many years of musical experience do you have (self-instruction included)?

12. How SOCIAL are you generally speaking?

Do NOT AGREE at all (1) (2) (3) (4) (5) (6) Totally AGREE (7)

I am very social

13. On average, how TECHNICALLY SKILLED are you with COMPUTERS?

Do NOT AGREE at all (1) (2) (3) (4) (5) (6) Totally AGREE (7)

I am technically skilled with computers

**Section 3**

**Music making BEFORE lockdown**

*How did you you play music with others before the lockdown, TEACHING AND THERAPY EXCLUDED?*

14. On average, how often did you play/sing music with others, while all being PHYSICALLY PRESENT, BEFORE the lockdown?

- More than once a week
- About once a week
- About once every two weeks
- About once a month
- Less than once a month

15. Did you ever play/sing LIVE (=in real-time) music with others through an ONLINE platform BEFORE the lockdown?

- Yes
- No *(redirected to question 19)*
- I don't know *(redirected to question 19)*

16.On average, how often did you play/sing LIVE music with others through an ONLINE platform BEFORE the lockdown?

- More than once a week
- About once a week
- About once every two weeks
- About once a month
- Less than once a month

17.What ONLINE PLATFORMS did you ever use to make LIVE music with others BEFORE the lockdown?

- JamKazam
- SoundJack
- Jamulus
- Ninjam
- JackTrip
- Messenger
- Skype
- Zoom
- Google Hangouts
- Microsoft Teams
- Starleaf
- Whereby
- Other…

18. To what extent did the MOST SUCCESSFUL PLATFORM you used BEFORE the lockdown enable you to achieve these items:

Do NOT AGREE at all (1) (2) (3) (4) (5) (6) Totally AGREE (7)

I successfully played live (=in real-time) music with others

It was fun to play music with others

It was fun to play music with others

I felt connected with (some of) the others while playing music

I reached a similar experience as when I would have played physically together with others

I successfully reached the goal I had in mind to achieve together with others

I was able to synchronize my performance with others

19. BEFORE the lockdown, to what extent did you feel held back by the required TECHNICAL CAPACITIES to work with these LIVE ONLINE PLATFORMS?

Do NOT AGREE at all (1) (2) (3) (4) (5) (6) Totally AGREE (7)

I felt held back by my TECHNICAL CAPACITIES to work with these platforms BEFORE the lockdown

20. Did you ever make use of OTHER METHODS to play/sing music with others BEFORE the lockdown? (Multiple answers possible)

- Yes, we recorded our parts separately and send these back and forth
- Yes, I played/jammed with others using pre-recorded videos/audio on Youtube/Spotify/...
- No
- I don't know
- Other…

21. BEFORE the lockdown, to what extent did you feel held back by the required TECHNICAL CAPACITIES to work with these OTHER METHODS?

Do NOT AGREE at all (1) (2) (3) (4) (5) (6) Totally AGREE (7)

I felt held back by my TECHNICAL CAPACITIES to work with these other methods BEFORE the lockdown

22. On average (physically, online, or other), to what extent did you ACTIVELY PURSUE playing music with others BEFORE the lockdown?

Do NOT AGREE at all (1) (2) (3) (4) (5) (6) Totally AGREE (7)

I actively pursued playing music with other BEFORE the lockdown

23. On average (physically, online, or other), to what extent did the SOCIAL CONNECTION with others play a part in your musical activities BEFORE the lockdown?

Do NOT AGREE at all (1) (2) (3) (4) (5) (6) Totally AGREE (7)

The SOCIAL CONNECTION with others played an important part in my musical activities BEFORE the lockdown

24. In order of importance, rank these reasons WHY (physically, online or otherwise) you played/sang music with others BEFORE the lockdown? (1= most important; drag to adjust order)

To have a good time

To feel connected with others

To improve my musical skills

To maintain/expand my network with other musicians

To express myself creatively/personally

To earn money

25. In what formation(s) did you play/sing (physically, online, or other) music with others BEFORE the lockdown? (e.g., band, choir, ...)

**Section 4**

**Playing music PHYSICALLY together DURING lockdown**

*How do you you play music with others while all being physically present during the lockdown, TEACHING AND THERAPY EXCLUDED?*

26. Did you already play/sing music with others, while all being PHYSICALLY PRESENT, DURING the lockdown?

- Yes
- No *(redirected to Section 5)*

27. HOW did you already play/sing music with others, while all being PHYSICALLY PRESENT, DURING the lockdown? (multiple answers possible)

- In the same room
- Not in the same room (e.g., with neighbours on the street/through the window)
- Other…

28.On average, how often do you play/sing music with others, while all being PHYSICALLY PRESENT, DURING the lockdown?

- More than once a week
- About once a week
- About once every two weeks
- About once a month
- Less than once a month

**Section 5**

**Playing music ONLINE LIVE with others DURING lockdown**

*How do you you play music 'live' (=in real-time) with others ONLINE (≠ making recordings) during the lockdown, TEACHING AND THERAPY EXCLUDED?*

29. Did you already play/sing LIVE (=in real-time) music with others through an ONLINE platform DURING the lockdown?

- Yes
- No *(redirected to question 35)*

30. On average, how often do you play/sing LIVE music with others through an ONLINE platform DURING the lockdown?

- More than once a week
- About once a week
- About once every two weeks
- About once a month
- Less than once a month

31.What ONLINE PLATFORMS did you already use to make LIVE music with others DURING the lockdown?

- JamKazam
- SoundJack
- Jamulus
- Ninjam
- JackTrip
- Messenger
- Skype
- Zoom
- Google Hangouts
- Microsoft Teams
- Starleaf
- Whereby
- Other…

32. With which one of the tested platforms do you have the BEST experience and explain WHY

33. With which one of the tested platforms do you have the WORST experience and explain WHY

34.To what extent enabled the MOST SUCCESSFUL PLATFORM you used DURING the lockdown you to achieve these items:

Do NOT AGREE at all (1) (2) (3) (4) (5) (6) Totally AGREE (7)

I successfully played live (=in real-time) music with others

I had fun making music with others

I felt connected with (some of) the others while making music

I reached a similar experience as when making music while physically present

I successfully reached the goal I had in mind to achieve together with others

I was able to synchronize my performance with others

*(redirected to question 36)*

35. Why did you NOT yet play/sing LIVE (=in real-time) music with others through an ONLINE platform DURING the lockdown? (Multiple possible answers)

- I did not feel a need to do so
- I know from personal experience that this does not work for me
- I heard from others that this does not work
- I did not know of its existence
- I do not have the time for it
- Other…

36. DURING the lockdown, to what extent do you feel held back by the required TECHNICAL CAPACITIES to work with these LIVE ONLINE PLATFORMS?

Do NOT AGREE at all (1) (2) (3) (4) (5) (6) Totally AGREE (7)

I feel held back by my TECHNICAL CAPACITIES to work with these online platforms DURING the lockdown

**Section 6**

**OTHER METHODS to play music with others DURING lockdown**

*How do you you play music using other methods during the lockdown, TEACHING AND THERAPY EXCLUDED?*

37. Did you already make use of OTHER METHODS to play/sing music with others DURING the lockdown? (e.g., records parts separately and send via mail)

- Yes
- No *(redirected to question 42)*

38. WHICH OTHER METHOD(S) did you already use to play/sing music with others DURING the lockdown?

- We recorded our parts separately and send these back and forth
- I played/jamed with others using pre-recorded videos/audio on Youtube/Spotify/...
- Other…

39. On average, how often do you play/sing music with others using OTHER METHODS DURING the lockdown?

- More than once a week
- About once a week
- About once every two weeks
- About once a month
- Less than once a month

40. With which one of the other methods do you have the BEST experience and explain WHY

41. To what extent did the MOST SUCCESSFUL OTHER METHOD you used DURING the lockdown enable you to achieve these items:

Do NOT AGREE at all (1) (2) (3) (4) (5) (6) Totally AGREE (7)

I successfully played live (=in real-time) music with others

I had fun making music with others

I felt connected to (some of) the others while making music

I reached a similar experience as when making music while physically present

I successfully reached the goal I had in mind to achieve together with others

I was able to synchronize my performance with others

*(redirected to question 43)*

42. Why did you NOT make use of OTHER METHODS to play/sing music with others DURING the lockdown? (Multiple possible answers)

- I did not feel a need to do so
- I know from personal experience that this does not work for me
- I heard from others that this does not work
- I did no know of its existence
- I do not have time for it
- Other…

43.DURING the lockdown, to what extent do you feel held back by the required TECHNICAL CAPACITIES to work with these OTHER METHODS?

Do NOT AGREE at all (1) (2) (3) (4) (5) (6) Totally AGREE (7)

I feel held back by my TECHNICAL CAPACITIES to work with these other methods DURING the lockdown

**Section 7**

**Making music with others DURING the lockdown: GENERAL**

*How do you you play music in general (physically, live online and/or via other methods) with others during the lockdown, TEACHING AND THERAPY EXCLUDED?*

44. Do you feel a GREATER NEED to play/sing music with other DURING the lockdown than before?

- Yes *(redirected to question 45)*
- No *(redirected to question 46)*
- Other…

45. Why do you feel a GREATER NEED to play/sing music with other DURING the lockdown than before? (Multiple possible answers)

- Playing/singing music together helps me to deal with the SITUATION and/or STRESS
- Playing/singing music together helps me to feel SOCIALLY CONNECTED to others
- Playing/singing music with others helps me to counter BOREDOM
- Other…

*(redirected to question 48)*

46. Why DON'T you feel a GREATER NEED to play/sing music with others DURING the lockdown than before?

- I feel about an EQUAL need to play/sing music with others *(redirected to question 48)*
- I feel LESS need to play/sing music with others *(redirected to question 47)*

47. Why do you feel LESS need to play/sing music with others?

48. Do you think you play/sing music MORE or LESS OFTEN with others DURING the lockdown than before?

- I play/sing LESS with OTHERS than before the lockdown *(redirected to question 49)*
- I play/sing about AS OFTEN with OTHERS than before the lockdown *(redirected to question 51)*
- I play/sing MORE with OTHERS than before the lockdown *(redirected to question 50)*

49. WHY do you play/sing LESS music with others than before the lockdown?

*(redirected to question 51)*

50. WHY do you play/sing MORE music with others than before the lockdown?

51. On average, to what extent do you ACTIVELY PURSUE playing music with others DURING the lockdown?

Do NOT AGREE at all (1) (2) (3) (4) (5) (6) Totally AGREE (7)

I actively pursue playing music with other DURING the lockdown

52. To what extent do you MISS making music with others DURING the lockdown as before the lockdown?

Do NOT AGREE at all (1) (2) (3) (4) (5) (6) Totally AGREE (7)

I MISS making music with others as before

53. To what extent does a SOCIAL CONNECTION with others play a part in your musical activities DURING the lockdown?

Do NOT AGREE at all (1) (2) (3) (4) (5) (6) Totally AGREE (7)

The SOCIAL CONNECTION with others plays an important role in my musical activities DURING the lockdown

54. In order of importance, rank these reasons WHY (physically, online or otherwise) you play/sing music with others DURING the lockdown? (1= most important; drag to adjust order)

To have a good time

To feel connected with others

To improve my musical skills

To maintain/expand my network with other musicians

To express myself creatively/personally

To earn money

55. In what formation(s) do you play/sing (physically, online, or other) music with others DURING the lockdown? (e.g., band, choir, ...)

56. Has the musical network with which you play/sing music changed since the lockdown?

Become a lot smaller (1) (2) (3) Stayed approximately the same (4) (5) (6) Become a lot bigger (7)

Since the lockdown, my musical network of people I play/sing with has...

**Section 8**

Thank you!

Thank you for partaking in our study. If you want to share comments or remarks, you can do so here.

If you would like to be informed about the results of the study, please contact Kelsey Onderdijk (kelsey.onderdijk@ugent.be). Please do not leave your contact info in the section below.

57. Possible remarks or comments

# Supplementary Material 2: Administered survey in Dutch

**Samen muziek maken: offline en online**

Hartelijk dank voor je interesse in deze studie.

Als onderzoekers in de musicologie aan de Universiteit Gent zijn we bijzonder geïnteresseerd in hoe mensen muziek maken met anderen. Het gaat hierbij om de periode voor en/of tijdens de huidige lockdown, met een specifieke focus op het gebruik van bestaande online platformen.

In deze survey stellen we je enkele vragen over je persoonlijke/muzikale achtergrond en je muzikaal gedrag voor en tijdens de lockdown. Deelname duurt max. 20 minuten.

Anonimiteit en vertrouwelijkheid worden gegarandeerd doorheen het hele proces van dataverzameling, -verwerking, -bewaring en rapportering van de data.

Gelieve contact met ons op te nemen indien u verdere vragen/opmerkingen heeft.

Contact:

Kelsey Onderdijk (Kelsey.Onderdijk@ugent.be)

**Section 1**

**Geïnformeerde toestemming**

Indien je beslist om aan deze studie deel te nemen, vragen we om je goedkeuring betreffende onderstaande items:

Ik verklaar dat ik wens te participeren in een vragenlijst van de Universiteit Gent en dat ...

(1) ik de informatie aangaande de aard van de studie en de data die wordt verzameld gelezen heb, en de mogelijkheid kreeg om extra vragen te stellen (zie contact info op de eerste en laatste pagina)

(2) ik volledig vrijwillig aan deze studie deel neem

(3) ik toestemming verleen om mijn resultaten anoniem te verwerken, bewaren en rapporteren

(4) ik me ervan bewust ben dat ik mijn deelname op elk moment kan stopzetten

(5) ik me realiseer dat geen deelname of stopzetting van deelname op geen enkel wijze eventuele evaluatie en/of tutoring van de Universiteit Gent zal beïnvloeden

(6) ik een samenvatting van de onderzoeksbevinding kan verkrijgen op aanvraag (zie contact info op de eerste en laatste pagina)

1. Heb je bovenstaande uitspraken gelezen?

- Ja, ik geef hierbij mijn toestemming om volgens bovenstaande afspraken aan deze studie deel te nemen

**Section 2**

**Achtergrondinformatie**

2. Wat is je leeftijd?

3. Wat is je geslacht?

- Vrouw
- Man
- X
- Geen antwoord
- Other…

4. In welk land verblijf je momenteel?

5. In welk type huishouden leef je?

- Alleenwonend
- Koppel, zonder kind(eren)
- Koppel, met kind(eren)
- Alleenwonend met kind(eren)
- Met mijn ouder(s), familie, vrienden of kennissen
- Geen antwoord
- Other…

6. Wat is je hoogst behaalde opleidingsniveau? Als je momenteel studeert, kies dan het niveau dat je momenteel nastreeft

- Basis onderwijs
- Secundair onderwijs
- Professionele verdere vorming (MBO/7de jaar/...)
- Hoger onderwijs (Universiteit/Hogeschool/...)
- Post-universitair (PhD)
- Other…

7. Wat is je huidige werksituatie?

- Werkend, voltijds
- Werkend, minder dan voltijds
- Werkloos (al dan niet tijdelijk)
- Student
- Other…

8. Is je persoonlijke belangrijkste inkomensbron afhankelijk van je muzikale activiteiten (lesgeven inclusief)?

- Ja
- Neen
- Other…

9. Welk type formele/informele muziekopleiding kreeg je of krijg je momenteel?

- Muziekacademie
- Private lessen
- Zelfstudie (autodidact)
- Conservatorium
- Musicologie
- Ik kreeg nooit een muziekopleiding
- Other…

10. Welk(e) instrument(en) (zang inbegrepen) speel je momenteel?

11. In totaal, hoeveel jaren muziekopleiding heb je achter de rug (zelfstudie inbegrepen)?

12. Hoe SOCIAAL ben je in het algemeen?

Helemaal NIET akkoord (1) (2) (3) (4) (5) (6) Helemaal akkoord (7)

Ik ben erg sociaal

13. Gemiddeld gezien, hoe TECHNISCHE ONDERLEGD ben je wat COMPUTERS betreft?

Helemaal NIET akkoord (1) (2) (3) (4) (5) (6) Helemaal akkoord (7)

Ik ben technisch onderlegd wat computers betreft

**Section 3**

**Samen muziek maken VOOR de lockdown**

*Hoe speelde je muziek met anderen VOOR de lockdown, LESGEVEN & THERAPIE NIET INBEGREPEN?*

14. Gemiddeld, hoe vaak speelde/zong je muziek met anderen, terwijl iedereen FYSIEK AANWEZIG was, VOOR de lockdown?

- Meer dan eenmaal per week
- Ongeveer eenmaal per week
- Ongeveer om de twee weken
- Ongeveer eenmaal per maand
- Minder dan eenmaal per maand

15. Speelde/zong je ooit LIVE (=in real-time) muziek met anderen via een ONLINE platform VOOR de lockdown?

- Ja
- Neen *(redirected to question 19)*
- Ik weet het niet *(redirected to question 19)*

16. Gemiddeld, hoe vaak speelde/zong je LIVE muziek met anderen via een ONLINE platform VOOR de lockdown

- Meer dan eenmaal per week
- Ongeveer eenmaal per week
- Ongeveer om de twee weken
- Ongeveer eenmaal per maand
- Minder dan eenmaal per maand

17.Welke ONLINE PLATFORMEN gebruikte je ooit al om LIVE muziek te maken met anderen VOOR de lockdown?

- JamKazam
- SoundJack
- Jamulus
- Ninjam
- JackTrip
- Messenger
- Skype
- Zoom
- Google Hangouts
- Microsoft Teams
- Starleaf
- Whereby
- Other…

18. In hoeverre stelde het MEEST SUCCESVOLLE PLATFORM dat je VOOR de lockdown gebruikte je in staat om deze items te bereiken:

Helemaal NIET akkoord (1) (2) (3) (4) (5) (6) Helemaal akkoord (7)

Ik kon met succes live (=in real-time) muziek spelen met anderen

Het was plezierig om muziek te maken met anderen

Ik voelde me met (sommige van) de anderen verbonden tijdens het musiceren

Ik kon een gelijkaardige ervaring bereiken als wanneer ik fysiek met anderen zou samen spelen

Ik bereikte met succes het doel dat ik voor ogen had om samen met de anderen te bereiken

Ik was in staat om mijn spel met anderen te synchroniseren

19. In welke mate voelde je je VOOR de lockdown tegengehouden door de vereiste TECHNISCHE CAPACITEITEN om met deze LIVE ONLINE PLATFORMEN aan de slag te gaan?

Helemaal NIET akkoord (1) (2) (3) (4) (5) (6) Helemaal akkoord (7)

Ik voelde me tegengehouden door mijn TECHNISCHE CAPACITEITEN om met deze platformen te werken VOOR de lockdown

20. Maakte je reeds gebruik van ANDERE METHODES om muziek te spelen/zingen met anderen VOOR de lockdown? (Meerdere antwoorden mogelijk)

- Ja, we namen onze delen apart op en stuurden de opnames heen en terug
- Ja, ik speelde/jamde met anderen via op voorhand opgenomen video's/audio op YouTube/Spotify/...
- Neen
- Ik weet het niet
- Other…

21. In welke mate voelde je je VOOR de lockdown tegengehouden door de vereiste TECHNISCHE CAPACITEITEN om met deze ANDERE METHODES aan de slag te gaan?

Helemaal NIET akkoord (1) (2) (3) (4) (5) (6) Helemaal akkoord (7)

Ik voelde me tegengehouden door mijn TECHNISCHE CAPACITEITEN om met deze andere methodes te werken VOOR de lockdown

22. In het algemeen (fysiek, online of anders), in welke mate STREEFDE JE ACTIEF NA om met anderen te musiceren VOOR de lockdown?

Helemaal NIET akkoord (1) (2) (3) (4) (5) (6) Helemaal akkoord (7)

Ik streefde actief na om met anderen te musiceren VOOR de lockdown

23. In het algemeen (fysiek, online of anders), in welke mate speelde de SOCIALE VERBONDENHEID met anderen een rol in je muzikale activiteiten VOOR de lockdown?

Helemaal NIET akkoord (1) (2) (3) (4) (5) (6) Helemaal akkoord (7)

De SOCIALE VERBONDENHEID met anderen speelde een belangrijke rol in mijn muzikale activiteiten VOOR de lockdown

24. Rangschik in volgorde van belangrijkheid deze redenen WAAROM je (fysiek, online of anders) muziek speelde/zong met anderen VOOR de lockdown? (1=meest belangrijk; versleep om volgorde te veranderen)

Om een goede tijd te beleven

Om me met anderen verbonden te voelen

Om mijn muzikale vaardigheden te verbeteren

Om mijn netwerk met andere muzikanten te onderhouden/uit te breiden

Om mezelf creatief/persoonlijk uit te drukken

Om geld te verdienen

25. In welke samenstelling(en) speelde/zong (fysiek, online of anders) je muziek met anderen VOOR de lockdown? (vb. band, koor, ...)

**Section 4**

**FYSIEK samen muziek maken TIJDENS lockdown**

*Hoe speelde je in levende lijve muziek met anderen tijdens de lockdown, LESGEVEN & THERAPIE NIET INBEGREPEN?*

26. Speelde/zong je reeds muziek met anderen, terwijl iedereen FYSIEK AANWEZIG was, TIJDENS de lockdown?

- Ja
- Neen *(redirected to Section 5)*

27. HOE speelde/zong je reeds muziek met anderen, terwijl iedereen FYSIEK AANWEZIG was, TIJDENS de lockdown? (meerdere antwoorden mogelijk)

- In dezelfde ruimte
- Niet in dezelfde ruimte (vb. op straat met buren/door het raam)
- Other…

28. Gemiddeld, hoe vaak speel/zing je muziek met anderen, terwijl iedereen FYSIEK AANWEZIG is, TIJDENS de lockdown?

- Meer dan eenmaal per week
- Ongeveer eenmaal per week
- Ongeveer om de twee weken
- Ongeveer eenmaal per maand
- Minder dan eenmaal per maand

**Section 5**

**ONLINE LIVE samen muziek maken TIJDENS lockdown**

*Hoe musiceer je 'live' (= in real-time) online en met anderen (≠ opnames maken) tijdens de lockdown, LESGEVEN & THERAPIE NIET INBEGREPEN?*

29. Speelde/zong je TIJDENS de lockdown reeds LIVE (=in real-time) muziek met anderen via een ONLINE platform?

- Ja
- Neen *(redirected to question 35)*

30. Gemiddeld, hoe vaak speel/zing je reeds LIVE muziek met anderen via een ONLINE platform TIJDENS de lockdown?

- Meer dan eenmaal per week
- Ongeveer eenmaal per week
- Ongeveer om de twee weken
- Ongeveer eenmaal per maand
- Minder dan eenmaal per maand

31.Welke ONLINE PLATFORMEN gebruikte je reeds om LIVE muziek te maken met anderen TIJDENS de lockdown?

- JamKazam
- SoundJack
- Jamulus
- Ninjam
- Messenger
- Skype
- Zoom
- Google Hangouts
- Microsoft Teams
- Starleaf
- Whereby
- Other…

32. Met welk(e) van de uitgeteste platformen heb je de BESTE ervaring en leg uit WAAROM

33. Met welk(e) van de uitgeteste platformen heb je de SLECHTSTE ervaring en leg uit WAAROM

34. In hoeverre stelde het MEEST SUCCESVOLLE PLATFORM dat je TIJDENS de lockdown gebruikte je in staat om deze items te bereiken:

Helemaal NIET akkoord (1) (2) (3) (4) (5) (6) Helemaal akkoord (7)

Ik kon met succes live (=in real-time) muziek spelen met anderen

Het was plezierig om muziek te maken met anderen

Ik voelde me met (sommige van) de anderen verbonden tijdens het musiceren

Ik kon een gelijkaardige ervaring bereiken als wanneer ik fysiek met anderen zou samen spelen

Ik bereikte met succes het doel dat ik voor ogen had om samen met de anderen te bereiken

Ik was in staat om mijn spel met anderen te synchroniseren

*(redirected to question 36)*

35. Waarom speelde/zong je TIJDENS de lockdown nog NIET LIVE (=in real-time) muziek met anderen via een ONLINE platform? (Meerdere antwoorden mogelijk)

- Ik had hier geen behoefte aan
- Ik weet uit persoonlijke ervaring dat dit voor mij niet werkt
- Ik hoorde van anderen dat dit niet werkt
- Ik wist niet van het bestaan af
- Ik heb er geen tijd voor
- Other…

36. In welke mate voel je je TIJDENS de lockdown tegengehouden door de vereiste TECHNISCHE CAPACITEITEN om met deze LIVE ONLINE PLATFORMEN aan de slag te gaan?

Helemaal NIET akkoord (1) (2) (3) (4) (5) (6) Helemaal akkoord (7)

Ik voel me tegengehouden door mijn TECHNISCHE CAPACITEITEN om met deze platformen te werken TIJDENS de lockdown

**Section 6**

**ANDERE METHODES om samen muziek te maken TIJDENS lockdown**

*Hoe musiceer je aan de hand van andere methodes met anderen tijdens de lockdown, LESGEVEN & THERAPIE NIET INBEGREPEN?*

37. Maakte je reeds gebruik van ANDERE METHODES om muziek te spelen/zingen met anderen TIJDENS de lockdown? (vb. partijen apart opnemen en via mail doorsturen)

- Ja
- Neen *(redirected to question 42)*

38.WELKE ANDERE METHODE(S) gebruikte je reeds om muziek te spelen/zingen met anderen TIJDENS de lockdown?

- We namen onze aparte delen op en sturen de opnames heen en terug
- Ik speelde/jamde met anderen via op voorhand opgenomen video's/audio op YouTube/Spotify/...
- Other…

39. Gemiddeld, hoe vaak speel/zing je muziek met anderen via deze ANDERE METHODE(S) TIJDENS de lockdown?

- Meer dan eenmaal per week
- Ongeveer eenmaal per week
- Ongeveer om de twee weken
- Ongeveer eenmaal per maand
- Minder dan eenmaal per maand

40.Met welk(e) andere methode(s) heb je de BESTE ervaring en leg uit WAAROM

41. In hoeverre stelde de MEEST SUCCESVOLLE ANDERE METHODE die je TIJDENS de lockdown gebruikte je in staat om deze items te bereiken:

Helemaal NIET akkoord (1) (2) (3) (4) (5) (6) Helemaal akkoord (7)

Ik kon met succes live (=in real-time) muziek spelen met anderen

Het was plezierig om muziek te maken met anderen

Ik voelde me met (sommige van) de anderen verbonden tijdens het musiceren

Ik kon een gelijkaardige ervaring bereiken als wanneer ik fysiek met anderen zou samen spelen

Ik bereikte met succes het doel dat ik voor ogen had om samen met de anderen te bereiken

Ik was in staat om mijn spel met anderen te synchroniseren

*(redirected to question 43)*

42. Waarom maakte je nog GEEN gebruik van ANDERE METHODES om muziek te spelen/zingen met anderen TIJDENS de lockdown? (Meerdere antwoorden mogelijk)

- Ik had hier geen behoefte aan
- Ik weet uit persoonlijke ervaring dat dit voor mij niet werkt
- Ik hoorde van anderen dat dit niet werkt
- Ik wist niet van het bestaan af
- Ik heb er geen tijd voor
- Other…

43. In welke mate voel je je TIJDENS de lockdown tegengehouden door de vereiste TECHNISCHE CAPACITEITEN om met deze ANDERE METHODES aan de slag te gaan?

Helemaal NIET akkoord (1) (2) (3) (4) (5) (6) Helemaal akkoord (7)

Ik voel me tegengehouden door mijn TECHNISCHE CAPACITEITEN om met deze andere methodes te werken TIJDENS de lockdown

**Section 7**

**Samen muziek maken TIJDENS lockdown: ALGEMEEN**

*Hoe musiceer je in het algemeen (fysiek, live online en/of via andere methodes) muziek met anderen tijdens de lockdown, LESGEVEN & THERAPIE NIET INBEGREPEN?*

44. Voel je een GROTERE DRANG om muziek te spelen/zingen met anderen TIJDENS de lockdown dan voorheen?

- Ja *(redirected to question 45)*
- Neen *(redirected to question 46)*
- Other…

45. Waarom voel je een GROTERE DRANG om muziek te spelen/zingen met anderen TIJDENS de lockdown dan voorheen? (Meerdere antwoorden mogelijk)

- Samen musiceren helpt me om met de SITUATIE en/of STRESS OM TE GAAN
- Samen musiceren helpt me om me SOCIAAL VERBONDEN met anderen te VOELEN
- Samen musiceren helpt me om de VERVELING TEGEN TE GAAN
- Other…

*(redirected to question 48)*

46. Waarom voel je GEEN GROTERE DRANG om muziek te spelen/zingen met anderen TIJDENS de lockdown dan voorheen?

- Ik voelde ongeveer EVENVEEL drang om met anderen muziek te spelen/zingen *(redirected to question 48)*
- Ik voelde MINDER drang om met anderen muziek te spelen/zingen *(redirected to question 47)*

47. Waarom voelde je MINDER drang om met anderen muziek te spelen/zingen?

48. Speel/zing je volgens jou MEER of MINDER VAAK muziek met anderen TIJDENS de lockdown dan voorheen?

- Ik speel/zing MINDER VAAK muziek MET ANDEREN dan voor de lockdown *(redirected to question 49)*
- Ik speel/zing ongeveer EVEN VAAK muziek MET ANDEREN als voor de lockdown *(redirected to question 51)*
- Ik speel/zing VAKER muziek MET ANDEREN dan voor de lockdown *(redirected to question 50)*

49. WAAROM speel/zing je MINDER VAAK muziek met anderen dan voor de lockdown?

*(redirected to question 51)*

50. WAAROM speel/zing je VAKER muziek met anderen dan voor de lockdown?

51. In welke mate STREEF JE ACTIEF NA om met anderen te musiceren TIJDENS de lockdown?

Helemaal NIET akkoord (1) (2) (3) (4) (5) (6) Helemaal akkoord (7)

Ik streef actief na om met anderen te musiceren TIJDENS de lockdown

52. In welke mate MIS JE HET om samen muziek te spelen TIJDENS de lockdown zoals voor de lockdown?

Helemaal NIET akkoord (1) (2) (3) (4) (5) (6) Helemaal akkoord (7)

Ik MIS HET om samen muziek te spelen zoals voorheen

53. In welke mate speelt een SOCIALE VERBONDENHEID met anderen een rol in je muzikale activiteiten TIJDENS de lockdown?

Helemaal NIET akkoord (1) (2) (3) (4) (5) (6) Helemaal akkoord (7)

De SOCIALE VERBONDENHEID met anderen speelt een belangrijke rol in mijn muzikale activiteiten TIJDENS de lockdown

54. Rangschik in volgorde van belangrijkheid deze redenen WAAROM je (fysiek, online of anders) muziek speelt/zingt met anderen TIJDENS de lockdown? (1=meest belangrijk; versleep om volgorde te veranderen)

Om een goede tijd te beleven

Om me met anderen verbonden te voelen

Om mijn muzikale vaardigheden te verbeteren

Om mijn netwerk met andere muzikanten te onderhouden/uit te breiden

Om mezelf creatief/persoonlijk uit te drukken

Om geld te verdienen

55. In welke samenstelling(en) speel/zing (fysiek, online of anders) je muziek met anderen TIJDENS de lockdown? (vb. band, koor, ...)

56. Is het muzikale netwerk waarmee je samen speelt/zingt volgens jou veranderd sinds de lockdown?

Veel kleiner geworden (1) (2) (3) Ongeveer even groot gebleven (4) (5) (6) Veel groter geworden (7)

Sinds de lockdown is het muzikale netwerk waarmee ik samen speel/zing ...

**Section 8**

Dankjewel!

Hartelijk dank om aan deze studie deel te nemen. Indien je verdere opmerkingen over deze studie hebt, gelieve deze hier te delen.

Als je graag over de resultaten van deze studie geïnformeerd wordt, gelieve Kelsey Onderdijk (kelsey.onderdijk@ugent.be) te contacteren. Deel je contactgegevens aub niet in onderstaande sectie.

57. Mogelijke opmerkingen
